# Pulmonary valve infective endocarditis caused by *Mycobacterium abscessus*

**DOI:** 10.1186/s13019-023-02336-9

**Published:** 2023-07-07

**Authors:** Olajide A. Olatidoye, Sajjaad H. Samat, Kanhua Yin, Michael J. Bates

**Affiliations:** 1 Department of Cardiovascular Sciences, Brody School of Medicine, East Carolina University, 115 Heart Drive, Greenville, NC 27834 USA; 2 Department of Surgery, Michigan State University, Lansing, MI USA

## Abstract

Infective endocarditis caused by *Mycobacterium abscessus* is an uncommon event that, when it does occur, usually requires surgical valve replacement. The pulmonary valve is the least common heart valve involved in infective endocarditis. We present a rare case of isolated pulmonary valve endocarditis with *Mycobacterium abscessus* in a patient with recurrent sternal infections following repeated coronary artery bypass.

## Introduction

Right-sided infective endocarditis represents less than 10% of all infective endocarditis cases, with pulmonary valve involvement in less than 2% of cases [1]. Although rare, the incidence of right-sided infective endocarditis has steadily risen because of the increasing use of intravenous drugs [2]. In this report, we present a rare case of isolated pulmonary valve endocarditis attributable to *Mycobacterium abscessus* in a patient with recurrent sternal infections.

## Case report

A 54-year-old male with a history of coronary artery disease, type II diabetes mellitus, hypertension, and past surgical history of coronary artery bypass grafting (CABG) presented to his primary care physician with chest pain. He was found to have significant coronary artery restenosis and underwent a redo sternotomy and CABG. During his redo CABG, an intraoperative injury to the pulmonary artery outflow tract necessitated pulmonary valve replacement with a 27 mm homograft valve and repair of the pulmonary artery with patch angioplasty.

**Fig. 1:**
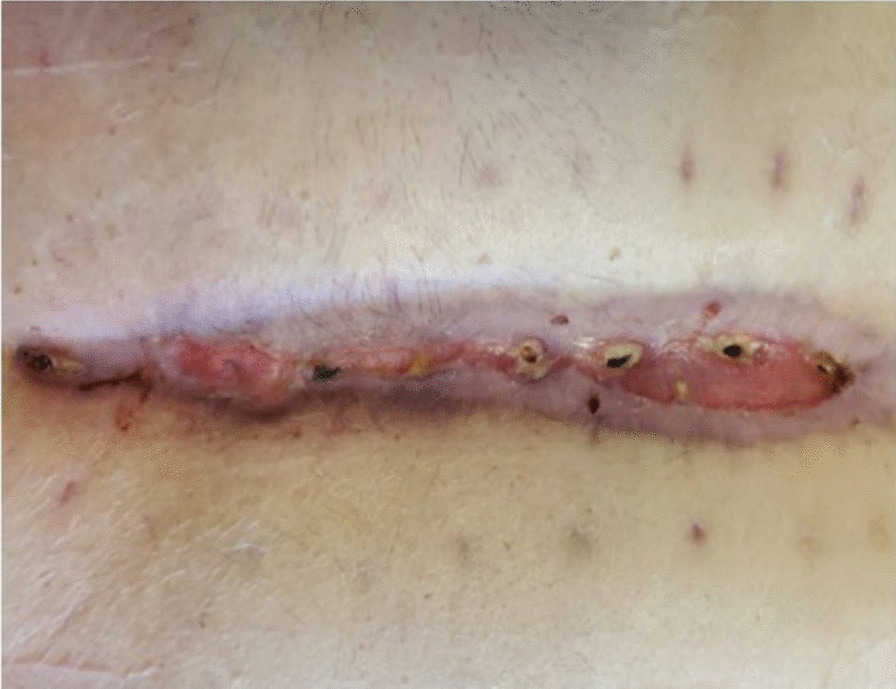
Sternal wound infection prior to transfer to our center

**Fig. 2:**
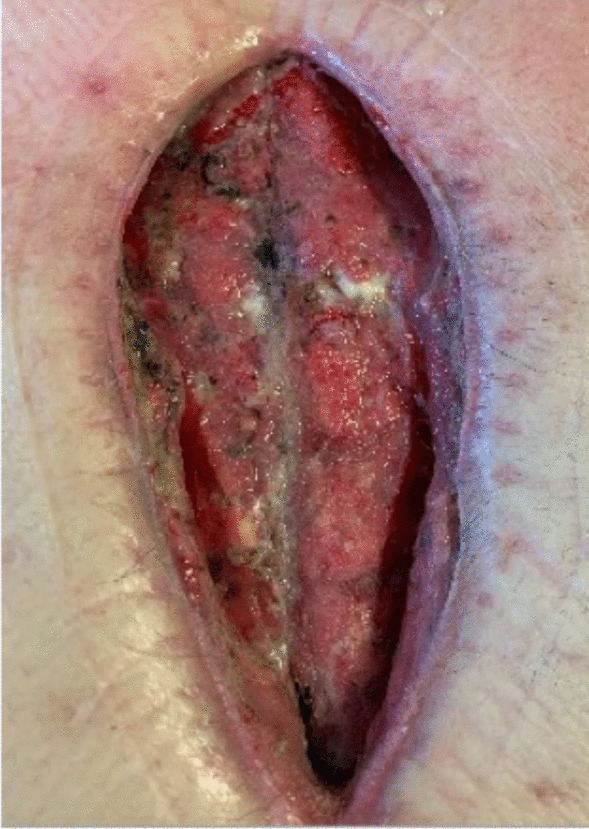
Sternal wound status post debridement and washout

Approximately one month following surgery, he presented with malodorous discharge from his sternal incision, requiring debridement with the placement of a Jackson-Pratt drain (Figs. 1 and 2). After sternal debridement, the patient was found to have persistent purulent drainage requiring additional six weeks of intravenous antibiotics along with the continuation of wound vacuum-assisted closure (VAC) changes. The patient underwent sternal wound washout with the removal of all sternal wires after completing the six-week regimen of antibiotics. Wound VAC therapy was continued, and his antibiotics were changed to Vancomycin and Piperacillin/Tazobactam.

The patient had blood cultures growing *Streptococcus dysgalactiae.* However, surgical wound cultures showed no growth. Due to persistent sternal infection and sepsis, he was transferred to our center and underwent another sternal wound debridement with bilateral pectoralis major flap coverage and drain placement. The drain was removed before his discharge on post-debridement day seven. After consultation with the infectious disease team, we de-escalated his antibiotics to penicillin for four additional weeks. Cultures from both blood and sternum were negative to date.

Although the patient remained on intravenous antibiotics, he developed a fever of 102 F, and the workup showed concern for an infected subpectoral seroma. The blood culture obtained at this time grew *Mycobacterium abscessus*. The patient underwent evacuation of the infected seroma with sternal debridement, and cultures of the seroma grew *Mycobacterium abscessus* and *Staphylococcus epidermidis.* The patient was subsequently started on sensitive antibiotics for *Mycobacterium abscessus.*

**Fig. 3:**
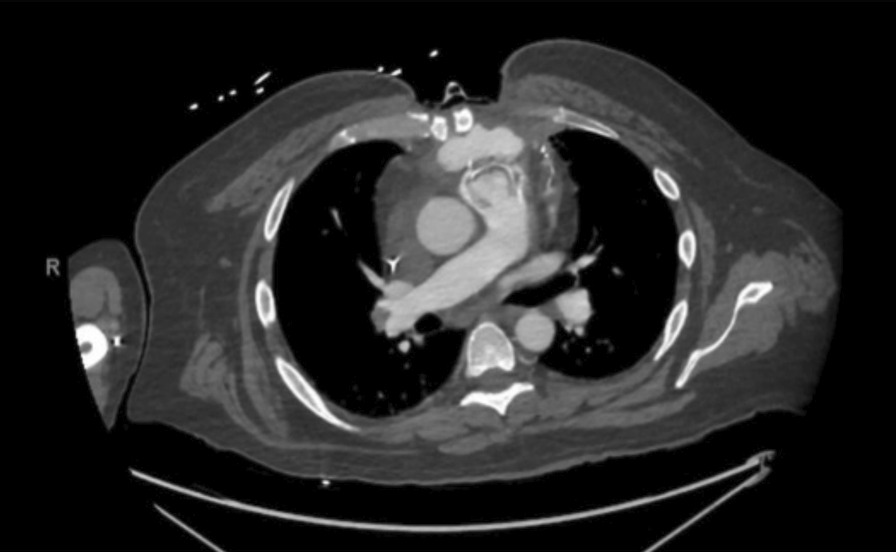
CT scan illustrating infected pulmonary artery pseudoaneurysm

However, he continued to have persistent intermittent fevers and underwent a radiological workup concerning an infected pulmonary artery pseudoaneurysm (Fig. 3). The patient was found to have an elevated procalcitonin level at 0.85 ng/mL. Given the complex clinical course, we discussed his case with our multidisciplinary team, and a week later, he underwent a redo sternotomy, resection of the pulmonary artery pseudoaneurysm with fresh pulmonary valve replacement, and chest wall reconstruction with a pectoralis muscle flap. Surgical cultures from the resected pulmonary artery pseudoaneurysm and the removed prosthetic pulmonary valve vegetations showed acid-fast bacilli. Wound cultures from the pulmonary valve also grew *Mycobacterium abscessus.* Based on sensitivities, he was started on amikacin, imipenem, and tigecycline. He was ultimately discharged to home on postoperative day 20 in stable condition. He has been followed up in the clinic and continued doing well two years after his most recent redo pulmonary valve surgery, with no evidence of acute or chronic infection.

## Discussion

Most infective endocarditis affects the left heart, and the most common organism is *Staphylococcus aureus* [3]. The right-sided endocarditis heart is much less common, and it usually involves the tricuspid valve when it does occur. Pulmonary valve endocarditis is even less common, accounting for less than 2% of all infective endocarditis cases [4]. Risk factors for the disease include intravenous drug use, central venous catheters, cyanotic congenital heart disease, and degenerative valve lesions. A 400-fold increase is observed in patients with prosthetic valves and a prior history of endocarditis [5]. In a retrospective study of 24 patients with pulmonary valve endocarditis, most had received prosthetic mechanical, autograft, or homograft valves [6].

*Mycobacterium abscessus* is a non-tuberculous, rapidly growing mycobacterium species that is exceptionally drug-resistant. It commonly infects indwelling medical devices such as catheters and prosthetic valves but is an uncommon agent of endocarditis. Through a literature search using PubMed/Medline, we identified 20 cases reporting infective endocarditis caused by *Mycobacterium abscessus*, including a review article summarizing ten cases published on or before 2008 and another ten sporadical cases published after 2008 (Table 1) [7–17]. Among these, eight (40%) involved prosthetic valve infections. The associated in-hospital mortality with endocarditis caused by *Mycobacterium abscessus* was very high (n = 12, 60%), despite surgery and prolonged antimicrobial therapy. Of note, only one case (5%) involved the pulmonary valve.

**Table 1:** Infective endocarditis caused by *Mycobacterium abscessus* published in the literature

Year	First author	Valve(s)	Prosthetic or native	In-hospital outcome
2008	Tsai [7]	3 isolated mitral, 2 isolated aortic, 2 isolated tricuspid, 1 mitral + aortic, 2 unknown	7 prosthetic, 3 native	7 died, 2 alive, 1 unknown
2010	Williamson [8]	Mitral	Native	Died
2010	Al-Benwan [9]	Mitral	Native	Died
2014	Garcia [10]	Mitral	Native	Died
2015	Mahajan [11]	Aortic	Native	Died
2015	Huth [12]	Tricuspid	Native	Alive
2017	Rodge [13]	Tricuspid	Native	Died
2017	Beatty [14]	Pulmonary	Prosthetic	Alive
2019	Sharma [15]	Aortic	Native	Alive
2021	Rahi [16]	Tricuspid + mitral	Native	Alive
2022	Maheshwarappa [17]	Aortic root	Native	Alive

Our patient had a sternal wound infection which may have seeded the infective endocarditis. Cultures from deep sternal wound infections often grow *Staphylococcus aureus*, coagulase-negative Staphylococcus species, and multiple mixed organisms [18]. Sternal mycobacterial infections are very uncommon, which further contributes to the uniqueness of this case.

## Conclusion

In summary, our patient represents the conjunction of several unusual clinical events, including isolated pulmonary valve infective endocarditis and *Mycobacterium abscessus* infection. This case highlights that mycobacterial infections may be difficult to identify, resistant to antibiotics and debridement therapy, and consequential in the outcome.

## Data Availability

The raw data are available on reasonable request due to privacy or ethical restrictions.
